# Assays to measure small molecule Hsp70 agonist activity *in vitro* and *in vivo*

**DOI:** 10.1016/j.ab.2024.115712

**Published:** 2024-11-09

**Authors:** Olivia Shapiro, Clara Woods, Amanda M. Gleixner, Sara Sannino, Marilyn Ngo, Michael D. McDaniels, Peter Wipf, Neil A. Hukriede, Christopher J. Donnelly, Jeffrey L. Brodsky

**Affiliations:** a Department of Neurobiology, LiveLikeLou Center for ALS Research, University of Pittsburgh, Pittsburgh, PA, 15261, USA; b Department of Cell Biology, Center for Integrative Organ Systems, University of Pittsburgh, Pittsburgh, PA, 15213, USA; c Department of Biological Science, University of Pittsburgh, Pittsburgh, PA, 15260, USA; d Department of Human Genetics, University of Pittsburgh School of Public Health, Pittsburgh, PA, 15213, USA; e Department of Chemistry, University of Pittsburgh, Pittsburgh, PA, 15260, USA

**Keywords:** Hsp70, Proteostasis, Molecular chaperone, Amyotrophic lateral sclerosis (ALS), Acute kidney injury (AKI), TDP-43

## Abstract

Hsp70 prevents protein aggregation and is cytoprotective, but sustained Hsp70 overexpression is problematic. Therefore, we characterized small molecule agonists that augment Hsp70 activity. Because cumbersome assays were required to assay agonists, we developed cell-based and *in vivo* assays in which disease-associated consequences of Hsp70 activation can be quantified. One assay uses an optogenetic system in which the formation of TDP-43 inclusions can be controlled, and the second assay employs a zebrafish model for acute kidney injury (AKI). These complementary assays will facilitate future work to identify new Hsp70 agonists as well as optimized agonist derivatives.

## 1. Introduction

The maintenance of the cellular proteome is overseen by protein homeostasis (proteostasis), which requires factors and pathways that sustain protein synthesis, folding, and function, and destroy toxic misfolded proteins. When the proteostasis network is overwhelmed, myriad human diseases arise [1]. Consequently, there is active interest in developing drugs to modulate nodes within the proteostasis network. Recent success in treating diseases associated with protein misfolding support the continued development of small molecules that target components of this network [2].

A key factor overseeing activities associated with proteostasis is the Hsp70 molecular chaperone. Hsp70 facilitates protein folding, orchestrates the delivery of misfolded proteins to degradative processes, decreases the concentration of protein aggregates and inclusions, facilitates post-translational modifications, and oversees protein transport [3]. Hsp70 is also anti-apoptotic [4]. Based on its cytoprotective effects, Hsp70 overexpression prevents toxicity associated with myriad diseases—many of which are linked to protein aggregation—in both cells and model organisms [5,6]. Unfortunately, increasing Hsp70 levels has a cost: prolonged Hsp70 elevation can trigger secondary unwanted outcomes on the pathways it regulates. Moreover, genetic strategies to specifically increase Hsp70 levels are currently unavailable for clinical applications.

As an alternate strategy, we posited that a small molecule Hsp70 activator would similarly protect the cellular proteome and show efficacy in disease models. By screening a collection of pyrimidone derivatives, we identified a compound, MAL1–271, that increases the ATPase activity of Hsp70 [7,8]. NMR spectroscopy identified the MAL1–271 binding site adjacent to where Hsp40 chaperones associate with Hsp70, suggesting MAL1–271 acts as an allosteric Hsp70 activator. Consistent with reduced protein aggregates/inclusions upon Hsp70 overexpression in neurodegenerative disease models, we showed that MAL1–271 reduces aggregation of both α-synuclein, which is linked to Parkinson’s disease [8,9], and a poly-glutamine repeat protein, which is a model for Huntington’s disease [10]. However, it was unknown whether MAL1–271 might reduce protein inclusions associated with other neurodegenerative diseases or diseases that affect non-neuronal tissues in which Hsp70 function is protective. An animal model in which potential effects on Hsp70 function might be identified was also desired.

To these ends, we developed two assays in which Hsp70 activation can be indirectly monitored in a quantitative fashion. First, we created a stable cell line in which expression of a Cry2-TDP-43 fusion protein can be controlled. In the presence of blue light, Cry2-TDP-43 forms inclusions, which mirror the behavior of TDP-43 inclusions in Amyotrophic Lateral Sclerosis (ALS, or Lou Gehrig’s disease) [11]. Second, we used a zebrafish model that—after administration of a nephrotoxin—recapitulates features associated with Acute Kidney Injury (AKI) [12–14].

## 2. Materials and methods

### 2.1. Chemicals

MAL1–271 and AMT-628–27 were synthesized as described, and purity and identity were validated [8]. Compounds were dissolved in DMSO to a final concentration of 10–30 mM, stored at −20 °C in single-use aliquots, and diluted to the appropriate concentration prior to use.

### 2.2. Cell line construction

The DNA construct containing Cry2 appended to TDP-43 and fused to mCherry (“optoTDP-43”) was described previously [11]. To create stable lines, HEK293 cells were seeded at 600,000 cells/well in a 6-well plate and grown to 90–100% confluency. The cells were then co-transfected with 600 ng of the PB-Cry2-TDP43-mch DNA construct and 120 ng of SB100X in a PCAG globin pA (transposase) DNA construct using Lipofectamine 2000 (Invitrogen), according to the manufacturer’s protocol. The cells were treated with 2.5 μg/mL puromycin, and antibiotic-resistant colonies were isolated, washed, and transferred to cloning discs (Fisher Scientific) soaked in trypsin-EDTA, grown to 90% confluency, passaged, and screened for protein expression after doxycycline hyclate (Dox; 500 ng/mL) addition.

### 2.3. Blue light-catalyzed formation of TDP43 inclusions

Blue light stimulated formation of Cry2-TDP-43-mCherry inclusions in the Dox-inducible stable HEK293 cells followed previous protocols in which Cry2-TDP-43-mCherry (“optoTDP43”) expression was examined in transiently transfected cells [11]. Briefly, optoTDP-43 cells in multi-well dishes were treated with 750 ng/mL doxycycline hyclate for 43 h at which point they were placed on an LED array (Amuza) for exposure to blue light at 1.75 mW/cm^2^ at 465 nm. Cells were exposed for 5 h (48 h total optoTDP-43 expression) under these conditions, washed with PBS, fixed, and processed for imaging [11].

Quantification of puncta and nuclear/cytoplasmic optoTDP-43 signal intensity was performed on maximum intensity projection images using the Nikon Elements GA3 automated analysis in a glass bottom 24 well plate. A minimum of 36 fields of view across three biological replicates (or only two biological replicates for untreated samples in the dark) were analyzed. We then quantified the number of cytoplasmic puncta per cell and the nuclear/cytoplasmic ratio of optoTDP-43 signal intensity. For both measures, a binary layer was first generated over cells expressing optoTDP-43 using intensity thresholding of the optoTDP-43 signal. Puncta were identified using a combination of intensity thresholding and bright spot detection. Upper and lower limits for intensity thresholds were manually set independently for each biological replicate, as fluorescence levels differed slightly between each replicate. Counts of optoTDP-43 expressing cells were taken using intensity thresholding and detection of nuclei (based on DAPI signal) under the binary layer of cells expressing optoTDP-43. Cytoplasmic puncta were defined as optoTDP-43 puncta detected outside the nuclear binary layer. To measure the optoTPD-43 nuclear/cytoplasmic ratio, the sum cytoplasmic signal was then quantified in the binary layer of cells expressing optoTDP-43 that had the nuclear binary subtracted. The sum intensity of optoTDP-43 within the nuclear binary was divided by the sum intensity of optoTDP-43 within the cytoplasmic binary, as defined above, for each field of view.

### 2.4. Zebrafish AKI model and RNA in situ hybridization

A zebrafish (*Danio rario*) model for AKI followed previous protocols, with modification, and was performed in 24- or 96-well dishes. In brief, 3 days post-fertilization zebrafish larvae were injected with gentamicin [12–14]. Larvae were anesthetized with 160 mg/mL tricaine (Sigma-Aldrich) and injected with 1 nL of gentamicin (Aspen Veterinary Resources) diluted in saline (for a final concentration of 7 ng/nL) via the common cardinal vein. After injection, larvae were incubated in 50 μg/mL penicillin/streptomycin in E3 medium. At 2 days post-injection, larvae were selected for edema and treated with either 0.5% DMSO, UPHD25 (4 μM), MAL1–271 (2 μM), or AMT-628–27 (2 μM). Larval survival was measured by the Kaplan–Meier estimator, using the Mantel-Cox log-rank test for significance (p value) and the Mantel-Haenszel Hazard Ratio [15]. UPHD25, an HDAC inhibitor previously shown to protect against kidney injury [16], was used as a positive control and was statistically significant in all survival assays. The *in situ* hybridization protocol was performed as described [17]. In all studies, larvae were arrayed and assayed daily from 5 to 9 days post-fertilization. Larvae were considered dead when a heartbeat and movement were absent after gentle prodding [14,16]. To measure the relative area/size of the fish and edema, we used the image analysis tool in Photoshop. All animal husbandry adhered to the National Institutes of Health Guide for the Care and Use of Laboratory Animals, and all zebrafish manipulated in these experiments were studied at a larval stage prior to sex differentiation.

## 3. Results and discussion

### 3.1. An Hsp70 agonist reduces toxic TDP43 inclusions

**Fig. 1.**
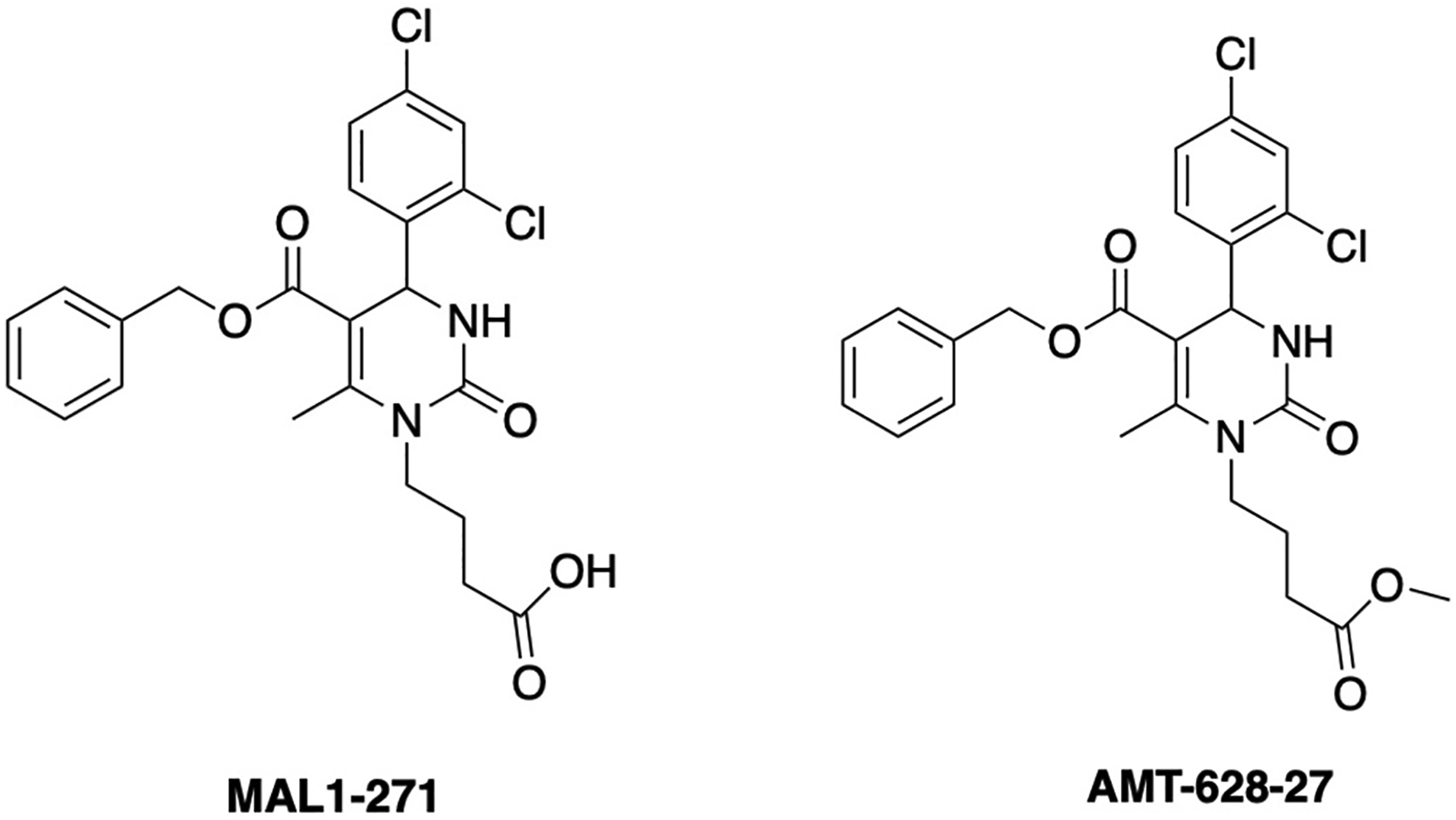
Chemical structures of Hsp70 agonists used in this study.

**Fig. 2.**
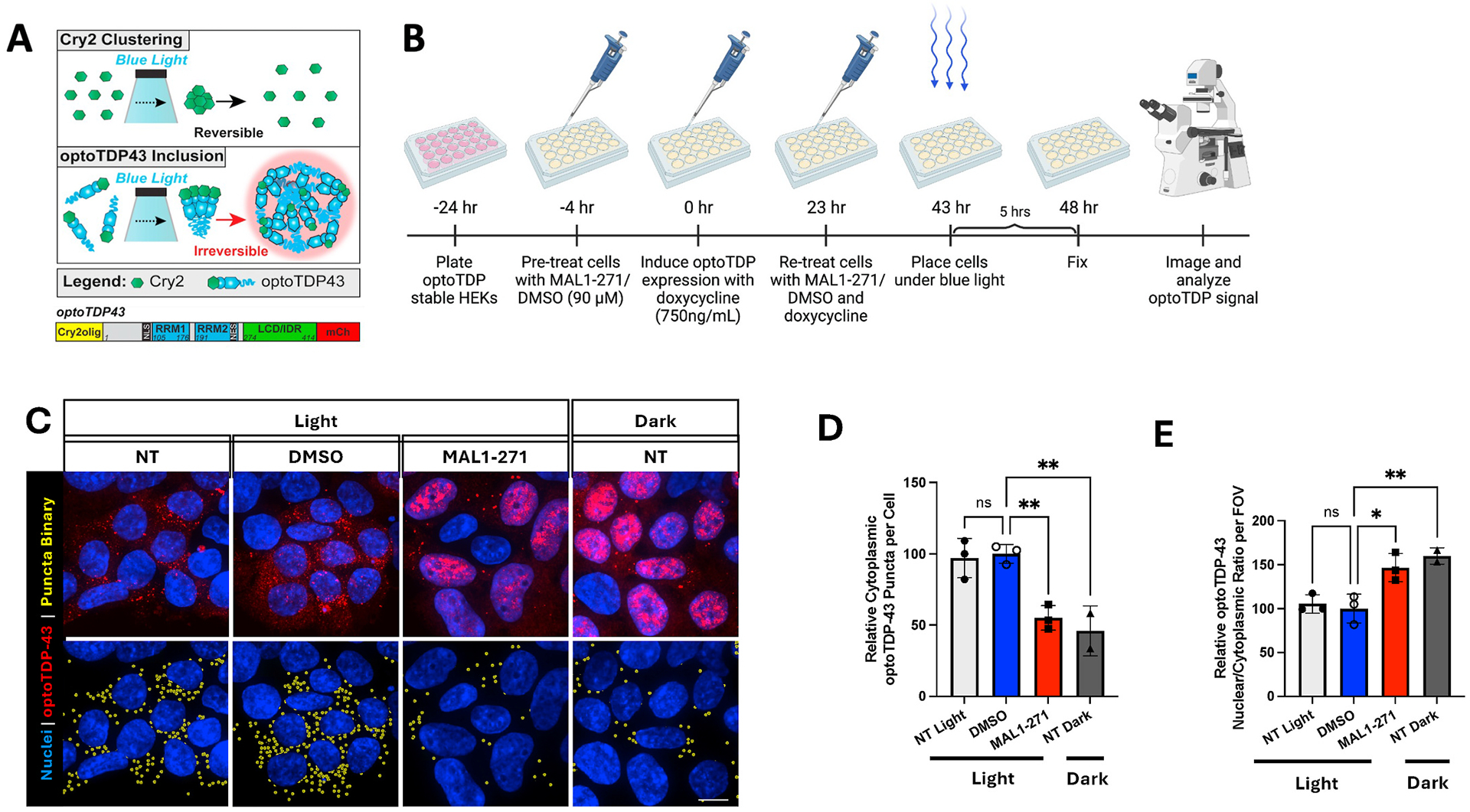
MAL1–271 reduces cytoplasmic Cry2-TDP43 puncta. **A.** Schematic of blue light-induced Cry2 clusters (top) and Cry2-TDP-43 irreversible inclusions (bottom), taken from Mann et al. [11]. A map of optoTDP43 is shown. **B.** Experimental timeline (image created with BioRender.com). **C**. Confocal microscopy images of HEK293 cells stably expressing Cry2-TDP-43-mCherry either not treated (NT), vehicle treated (DMSO), or treated with 90 μM MAL1–271, and in the presence or absence of blue light stimulation. Yellow binary layer overlay indicates TDP-43 puncta via bright spot and intensity thresholding in an automated analysis pipeline within Nikon Elements (see Materials and methods). **D.** Quantification of cytoplasmic puncta/cell in MAL1–271-treated cells at a final concentration of 90 μM (n = 3), normalized to the DMSO, light-stimulated condition. **E.** Quantification of the TDP-43 nuclear/cytoplasmic ratio. Statistical significance was determined by one-way ANOVA with Bonferroni’s multiple comparisons test. *p ≤ 0.05, **p ≤ 0.01. Please note that n refers to 3 independent experiments. For each experiment, at least 12 fields of view were analyzed per group. Data are shown as mean ± SD, n = 3.

A common neuropathological hallmark of ALS, frontotemporal dementia, and limbic-predominant age-related TDP-43 encephalopathy is the cytoplasmic mislocalization and formation of TDP-43 inclusions. Prior work indicated that Hsp70 co-localizes with TDP-43 and helps maintain its function and solubility [18]. To examine whether the MAL1–271 Hsp70 agonist [7–10] (Fig. 1) ameliorates pathophysiological features of TDP-43, we used a HEK293 cell model in which TDP-43 appended to mCherry along with a Cry2 optogenetic tag was expressed. Prior work showed that stable inclusions after blue light stimulation (which triggers Cry2 polymerization) recapitulate several ALS-like features (Fig. 2A) [11]. However, Cry2-TDP-43 expression was driven via transient transfection. Therefore, a doxycycline (Dox)-inducible cell line was constructed (see Materials and methods). We were also particularly interested in the ratio of cytoplasmic to nuclear inclusions since cytoplasmic inclusions are especially neurotoxic [19].

We pretreated HEK293 cells for 4 h with 90 μM MAL1–271 or an equivalent volume of DMSO before inducing Cry2-TDP-43 expression with Dox. Pre-treatment with MAL1–271 allows for activation of the Hsp70 pathway, which as shown in seminal studies in the chaperone field (see for example [20]) helps elicit protective responses. After 23 h, we reapplied compound, Cry2-TDP-43 polymerization was induced with blue light after 20 h, and the cells were fixed and imaged 5 h later (Fig. 2B). As shown in Fig. 2C, incubation of cells with MAL1–271 markedly reduced the number of cytoplasmic puncta by ~50%, which is similar to background levels (see yellow puncta, bottom panel; data quantified in Fig. 2D). This signal corresponding to puncta derives from the mCherry signal in the TDP-43 expression vector (see Materials and methods). As calculated in Fig. 2E, there was also an ~40 % increase in the relative amount of protein redistributed from the cytoplasm into the nucleus in the presence of the agonist (Fig. 2C, red puncta). Therefore, MAL1–271 decreases the accumulation and localization of the more toxic TDP-43 species. Together, these data are consistent with the effects of an Hsp70 agonist.

### 3.2. Hsp70 agonists decrease kidney injury in zebrafish

**Fig. 3.**
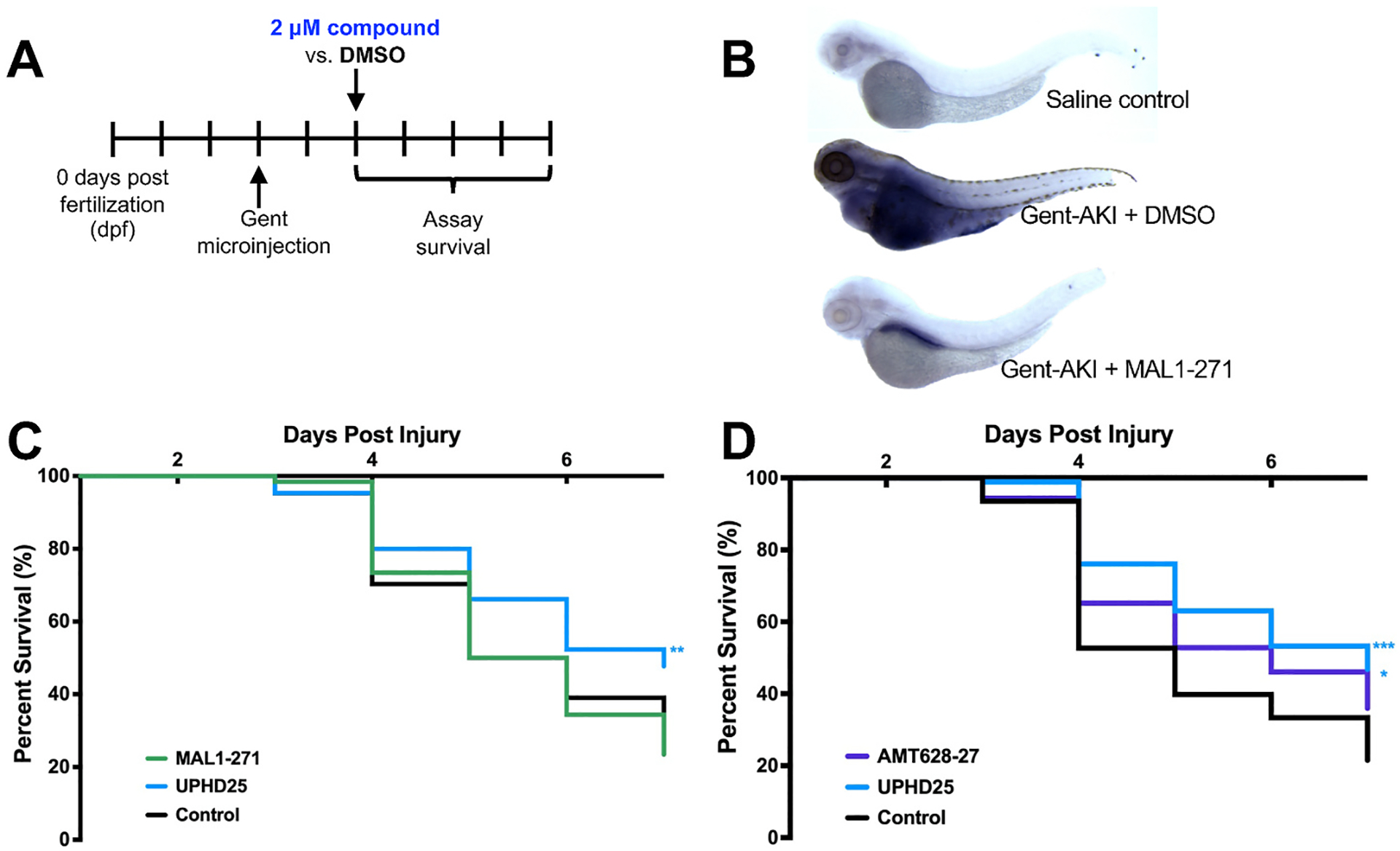
MAL1–271 treatment improves survival and reduces stress in a zebrafish AKI model. A. Experimental timeline. Uninjured, wild-type zebrafish larvae were injected with gentamycin at 3 days post-fertilization (dpf). At 2 days post-injection (dpi), edematous fish were selected and treated with 1 % DMSO vehicle (control), 2 μM MAL1–271, 2 μM AMT-628–27, or 4 μM UPHD25 (n = 4 experiments, 15–25 larvae per group). Survival was assessed from 3 dpi to 7 dpi. **B**. Representative embryos subject to the indicated treatment. After 48 h, embryos were processed for RNA *in situ* hybridization using an antisense probe against GCLM. **C.** Survival curve in the presence or absence of MAL1–271 treatment as compared to injured DMSO treated (control), and injured UPHD25 treated zebrafish. **D.** Survival curve in the presence or absence of AMT-628–27 treatment as compared to injured - DMSO treated (control) and injured - UPHD25 treated zebrafish. Data were analyzed as compounds that extend survival via the Kaplan–Meier estimator using a log-rank test (p value) between control (DMSO treated) injured larvae and compound treated larva: *p ≤ 0.05; **p ≤ 0.01; ***p ≤ 0.001. A Mantel-Haenszel hazard ratio, which expresses the chance (or hazard) of events occurring in the treatment as a ratio of events occurring in the control group, was also calculated. Compounds with a hazard ratio <1 indicate survival was improved in the treatment group versus the control group [15]. **C.** The hazard ratio for MAL1–217/control was 0.9989 and for UPHD25/control it was 0.5296. **D.** The hazard ratio for AMT-628–27/control was 0.6408 and for UPHD25/control it was 0.4434.

Based on these results—and the protective effects of MAL1–271 in other models [8–10]—we asked whether the compound was effective in an *in vivo* AKI model since Hsp70 overexpression protects renal function in rodents [21], and as noted above MAL1–271 is an allosteric activator and binds to the ATP-binding domain in Hsp70 *in vitro* [7]. In zebrafish embryos, the intravenous injection of a nephrotoxic antibiotic, gentamicin, damages renal progenitor cells, which triggers edema and death [22]. We therefore surmised that this indirect measure of gentamicin-induced cell stress might report on Hsp70 function. Thus, we followed the protocol outlined in Fig. 3A. To begin to investigate the effect of MAL1–217, *in situ* mRNA hybridization targeting a glutamate-cysteine ligase modifier subunit (GCLM), which is required for the rate limiting step in glutathione synthesis, was performed. Glutathione synthesis, which is reflective of GCLM levels, offsets the potentially catastrophic effects of reactive oxygen species on renal cell homeostasis, which in turn is associated with acute kidney injury [23]. For this initial study, we examined untreated, gentamicin injured, and gentamicin injured fish which were then administered 2 μM MAL1–271, a dose that is approximately, the LD_50_ for the compound (data not shown but see below). Compared to the saline-injected control, an enlarged body was apparent in the gentamicin-injured fish, which is consistent with edema, a condition seen in AKI (Fig. 3B) [22]. Yet, the extent of edema was reduced in the MAL1–271-treated fish. Based on simple image analysis (see Materials and methods), the body size of gentamicin-treated fish increased >5-fold, but the application of MAL1–271 decreased body size significantly so it was only ~10 % greater than the control. In addition, MAL1–271 treatment reduced GCLM expression almost to the level of the control. It is important to note, however, that *in situ* hybridizations are qualitative—not quantitative—measures of mRNA expression [24].

We next asked if MAL1–271 prolongs larval survival since we previously established survival as a marker of compound efficacy when examining a lead compound, UPHD25, which targets other cytoprotective effectors [16]. In this model, 2 μM MAL-1–271 had no effect on zebrafish lifespan, whereas UPHD25 increased survival (Fig. 3C). Because this might reflect toxicity (see above) and/or the need for an Hsp70 agonist with improved access to relevant tissues, we reasoned that esterification of the MAL1–271 free acid might be necessary. This line of reasoning is based on the fact that UPHD25 is the methyl ester prodrug of the active carboxylic acid, PTBA [16,22]. Indeed, when we applied a MAL1–271 derivative in which the free acid was esterified (AMT-628–27; Fig. 1) at a final concentration of at 2 μM and examined lifespan, effects on survival were now statistically significant (Fig. 3D).

## 4. Conclusions

By using new *in vitro* and *in vivo* models, we confirmed the ability of small molecule Hsp70 agonists to reduce protein aggregation and cell stress. Both models use multi-well dishes, which in the future might facilitate the identification of Hsp70 agonists in high throughput screens. Important secondary screens could include assays to ensure that any identified compounds from the screen lack cytotoxicity and the use of an integrated transgene containing a visual reporter for induction of an Hsp70-dependent stress pathway [25]. Although clinical applications of existing agonists would require improved drug-like properties, the compounds examined in this study can be used as positive controls in future screens.

## Data Availability

Data will be made available on request.
